# Dimethyl 4,4-diacetyl­hepta­nedioate

**DOI:** 10.1107/S1600536808043705

**Published:** 2009-01-08

**Authors:** Ling-hua Zhuang, Guo-wei Wang

**Affiliations:** a Department of Applied Chemistry, College of Science, Nanjing University of Technolgy, Nanjing 210009, People’s Republic of China; b Department of Light Chemical Engineering, College of Science, Nanjing University of Technolgy, Nanjing 210009, People’s Republic of China

## Abstract

The mol­ecule of the title dicarbonyl compound, C_13_H_20_O_6_, possesses approximate local twofold symmetry. In the crystal, inter­molecular C—H⋯O hydrogen bonds link the mol­ecules, generating a chain structure.

## Related literature

For general background, see: Kim *et al.* (2001 ▶); Chetia *et al.* (2004 ▶); Ranu & Banerjee (2005 ▶); Wang *et al.* (2008 ▶). For bond-length data, see: Allen *et al.* (1987 ▶).
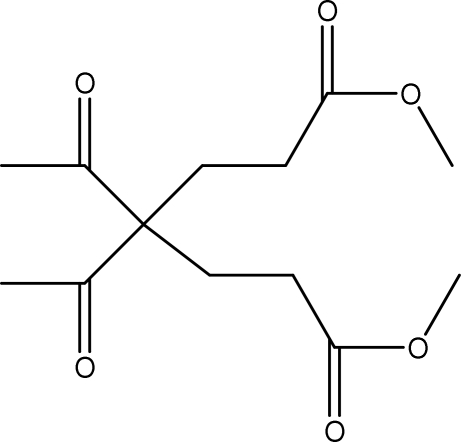


## Experimental

### 

#### Crystal data

C_13_H_20_O_6_
*M* *_r_* = 272.29
Monoclinic, 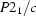

*a* = 11.402 (2) Å
*b* = 8.6910 (17) Å
*c* = 14.845 (3) Å
β = 107.35 (3)°
*V* = 1404.1 (5) Å^3^
*Z* = 4
Mo *K*α radiation
μ = 0.10 mm^−1^
*T* = 293 (2) K
0.30 × 0.20 × 0.10 mm

#### Data collection

Enraf–Nonius CAD-4 diffractometer
Absorption correction: ψ scan (North *et al.*, 1968 ▶) *T*_min_ = 0.970, *T*_max_ = 0.990
2531 measured reflections
2531 independent reflections
1783 reflections with *I* > 2σ(*I*)
3 standard reflections every 200 reflections intensity decay: 9%

#### Refinement

*R*[*F* ^2^ > 2σ(*F* ^2^)] = 0.065
*wR*(*F* ^2^) = 0.202
*S* = 1.01
2531 reflections
172 parameters
H-atom parameters constrained
Δρ_max_ = 0.38 e Å^−3^
Δρ_min_ = −0.25 e Å^−3^

### 

Data collection: *CAD-4 Software* (Enraf–Nonius, 1989 ▶); cell refinement: *CAD-4 Software*; data reduction: *XCAD4* (Harms & Wocadlo, 1995 ▶); program(s) used to solve structure: *SHELXS97* (Sheldrick, 2008 ▶); program(s) used to refine structure: *SHELXL97* (Sheldrick, 2008 ▶); molecular graphics: *SHELXTL* (Sheldrick, 2008 ▶); software used to prepare material for publication: *SHELXTL*.

## Supplementary Material

Crystal structure: contains datablocks global, I. DOI: 10.1107/S1600536808043705/sj2570sup1.cif

Structure factors: contains datablocks I. DOI: 10.1107/S1600536808043705/sj2570Isup2.hkl

Additional supplementary materials:  crystallographic information; 3D view; checkCIF report

## Figures and Tables

**Table 1:** Hydrogen-bond geometry (Å, °)

*D*—H⋯*A*	*D*—H	H⋯*A*	*D*⋯*A*	*D*—H⋯*A*
C4—H4*B*⋯O5^i^	0.97	2.58	3.418 (4)	145
C3—H3*B*⋯O2^ii^	0.97	2.55	3.500 (4)	165

Symmetry codes: (i) 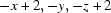; (ii) 
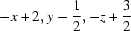
.

**Fig. 1.**
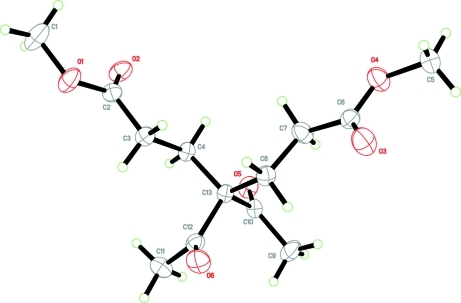
A view of the molecular structure of (I) showing the atom-numbering scheme and 30% displacement ellipsoids.

**Fig. 2.**
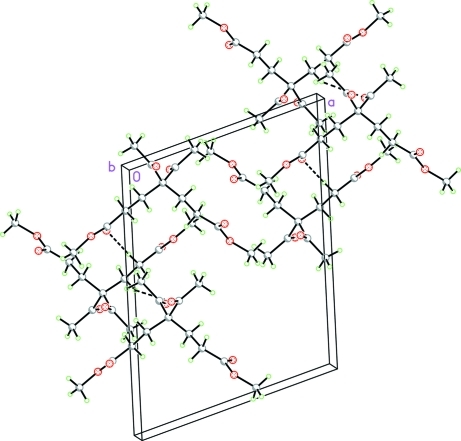
The crystal packing of (I) Intermolecular hydrogen bonds are shown as dashed lines.

## supplementary crystallographic information

### Comment

Dicarbonyl compounds represent an important class of starting materials used to increase the carbon number of organic compounds (Kim *et al.*, 2001). Some dicarbonyl compounds are useful for the synthesis of enantiomerically pure alcohols (Chetia *et al.*, 2004). Many dicarbonyl compounds have been synthesized with Michael Addition method using diethyl malonate as the starting mateiral, but only a few Michael Addition diadducts have been synthesized under normal condition (Ranu & Banerjee, 2005; Wang *et al.*, 2008). We are focusing our synthetic and structural studies on new products of Michael Addition diadducts from dicarbonyl compounds. We report here the crystal structure of the title dicarbonyl compound (I), Fig 1.

All bond lengths in the compound are within normal ranges (Allen *et al.*, 1987). The central C13 atom lies on a lies on a non-crystallographic pseudo twofold rotation axis. Intermolecular C—H···O hydrogen bond (Table 1, Fig.2) help to establish the one-dimensional supramolecular structure.

### Experimental

Acetylacetone (50 mmol), anhydrous potassium carbonate (100 mmol), tetrabutylammonium bromide (1 g) were dissolved in toluene (20 ml) and methyl acrylate (100 mmol) was slowly dropped to the mixture which was stirred for 24 h at 303–333 K, then 100 ml water was added. The organic layer was dried with magnesium sulfate and the solvent removed under vacuum to obtain the crude product, (I). Crystals suitable for X-ray diffraction were obtained by slow evaporation of an ethyl acetate solution. ^1^H NMR (CDCl_3_, δ, p.p.m.) 3.62(s, 6H), 2.23(m, 4H), 2.144 (m, 10H).

### Refinement

All H atoms were positioned geometrically, with C—H = 0.96 and 0.97 Å for methyl and methylene H atoms, and constrained to ride on their parent atoms, with *U*_iso_(H) = *xU*_eq_(C), where *x*= 1.5 for methyl H and *x* = 1.2 for methylene H atoms.

### Crystal data

**Table:**

C_13_H_20_O_6_	*F*(000) = 584
*M**_r_* = 272.29	*D*_x_ = 1.288 Mg m^−^^3^
Monoclinic, *P*2_1_/*c*	Mo *K*α radiation, λ = 0.71073 Å
Hall symbol: -P 2ybc	Cell parameters from 27 reflections
*a* = 11.402 (2) Å	θ = 8–15°
*b* = 8.6910 (17) Å	µ = 0.10 mm^−^^1^
*c* = 14.845 (3) Å	*T* = 293 K
β = 107.35 (3)°	Block, colourless
*V* = 1404.1 (5) Å^3^	0.30 × 0.20 × 0.10 mm
*Z* = 4	

### Data collection

**Table:**

Enraf–Nonius CAD-4 diffractometer	1783 reflections with *I* > 2σ(*I*)
Radiation source: fine-focus sealed tube	*R*_int_ = 0.0000
graphite	θ_max_ = 25.3°, θ_min_ = 1.9°
ω/2θ scans	*h* = −13→13
Absorption correction: ψ scan (North *et al.*, 1968)	*k* = 0→10
*T*_min_ = 0.970, *T*_max_ = 0.990	*l* = 0→17
2531 measured reflections	3 standard reflections every 200 reflections
2531 independent reflections	intensity decay: 9%

### Refinement

**Table:**

Refinement on *F*^2^	Primary atom site location: structure-invariant direct methods
Least-squares matrix: full	Secondary atom site location: difference Fourier map
*R*[*F*^2^ > 2σ(*F*^2^)] = 0.065	Hydrogen site location: inferred from neighbouring sites
*wR*(*F*^2^) = 0.202	H-atom parameters constrained
*S* = 1.00	*w* = 1/[σ^2^(*F*_o_^2^) + (0.1*P*)^2^ + 1.5*P*] where *P* = (*F*_o_^2^ + 2*F*_c_^2^)/3
2531 reflections	(Δ/σ)_max_ = 0.001
172 parameters	Δρ_max_ = 0.38 e Å^−^^3^
0 restraints	Δρ_min_ = −0.25 e Å^−^^3^

### Special details

**Table:**

Geometry. All e.s.d.'s (except the e.s.d. in the dihedral angle between two l.s. planes) are estimated using the full covariance matrix. The cell e.s.d.'s are taken into account individually in the estimation of e.s.d.'s in distances, angles and torsion angles; correlations between e.s.d.'s in cell parameters are only used when they are defined by crystal symmetry. An approximate (isotropic) treatment of cell e.s.d.'s is used for estimating e.s.d.'s involving l.s. planes.
Refinement. Refinement of *F*^2^ against ALL reflections. The weighted *R*-factor *wR* and goodness of fit *S* are based on *F*^2^, conventional *R*-factors *R* are based on *F*, with *F* set to zero for negative *F*^2^. The threshold expression of *F*^2^ > σ(*F*^2^) is used only for calculating *R*-factors(gt) *etc*. and is not relevant to the choice of reflections for refinement. *R*-factors based on *F*^2^ are statistically about twice as large as those based on *F*, and *R*- factors based on ALL data will be even larger.

### Fractional atomic coordinates and isotropic or equivalent isotropic displacement parameters (Å^2^)

**Table:**

	*x*	*y*	*z*	*U*_iso_*/*U*_eq_	
O1	1.1970 (2)	−0.2798 (3)	0.80271 (19)	0.0587 (7)	
O2	1.1274 (2)	−0.0419 (2)	0.80976 (18)	0.0552 (7)	
O3	0.5191 (2)	−0.3553 (3)	0.66776 (18)	0.0646 (7)	
O4	0.5065 (2)	−0.1212 (3)	0.60519 (17)	0.0585 (7)	
O5	0.7976 (2)	0.0496 (2)	0.94983 (17)	0.0506 (6)	
O6	0.8453 (2)	−0.4676 (2)	0.96422 (18)	0.0567 (7)	
C1	1.2919 (4)	−0.2184 (5)	0.7683 (3)	0.0710 (12)	
H1A	1.3430	−0.3006	0.7586	0.106*	
H1B	1.3407	−0.1472	0.8136	0.106*	
H1C	1.2556	−0.1660	0.7097	0.106*	
C2	1.1177 (3)	−0.1782 (3)	0.8194 (2)	0.0401 (7)	
C3	1.0191 (3)	−0.2557 (3)	0.8502 (2)	0.0408 (7)	
H3A	1.0564	−0.3225	0.9033	0.049*	
H3B	0.9697	−0.3190	0.7991	0.049*	
C4	0.9365 (2)	−0.1393 (3)	0.8785 (2)	0.0360 (7)	
H4A	0.9057	−0.0673	0.8269	0.043*	
H4B	0.9858	−0.0814	0.9324	0.043*	
C5	0.4002 (3)	−0.1660 (5)	0.5299 (3)	0.0597 (10)	
H5A	0.3724	−0.0805	0.4881	0.089*	
H5B	0.3361	−0.1974	0.5557	0.089*	
H5C	0.4211	−0.2501	0.4957	0.089*	
C6	0.5572 (3)	−0.2265 (4)	0.6703 (2)	0.0430 (7)	
C7	0.6632 (3)	−0.1613 (4)	0.7454 (2)	0.0513 (8)	
H7A	0.6340	−0.0785	0.7769	0.062*	
H7B	0.7214	−0.1179	0.7163	0.062*	
C8	0.7290 (3)	−0.2793 (3)	0.8186 (2)	0.0413 (7)	
H8A	0.7683	−0.3542	0.7887	0.050*	
H8B	0.6686	−0.3333	0.8410	0.050*	
C9	0.6618 (3)	−0.1291 (4)	0.9838 (3)	0.0582 (9)	
H9A	0.6315	−0.0401	1.0081	0.087*	
H9B	0.6907	−0.2033	1.0334	0.087*	
H9C	0.5967	−0.1734	0.9338	0.087*	
C10	0.7649 (3)	−0.0831 (4)	0.9467 (2)	0.0393 (7)	
C11	0.9581 (3)	−0.2844 (4)	1.0738 (2)	0.0539 (9)	
H11A	0.9808	−0.3720	1.1146	0.081*	
H11B	0.9172	−0.2102	1.1018	0.081*	
H11C	1.0305	−0.2389	1.0648	0.081*	
C12	0.8733 (3)	−0.3344 (3)	0.9802 (2)	0.0394 (7)	
C13	0.8265 (2)	−0.2094 (3)	0.9038 (2)	0.0341 (6)	

### Atomic displacement parameters (Å^2^)

**Table:**

	*U*^11^	*U*^22^	*U*^33^	*U*^12^	*U*^13^	*U*^23^
O1	0.0587 (14)	0.0424 (13)	0.0902 (18)	0.0057 (11)	0.0453 (14)	0.0031 (12)
O2	0.0663 (15)	0.0377 (13)	0.0743 (16)	−0.0010 (11)	0.0403 (13)	0.0039 (11)
O3	0.0630 (16)	0.0488 (15)	0.0686 (16)	−0.0126 (12)	−0.0008 (13)	0.0054 (12)
O4	0.0587 (15)	0.0523 (14)	0.0544 (14)	−0.0080 (11)	0.0015 (11)	0.0059 (11)
O5	0.0508 (13)	0.0329 (12)	0.0712 (16)	0.0012 (10)	0.0228 (11)	−0.0047 (10)
O6	0.0595 (15)	0.0344 (13)	0.0714 (16)	−0.0049 (10)	0.0123 (12)	0.0102 (11)
C1	0.065 (2)	0.063 (2)	0.105 (3)	−0.0007 (19)	0.055 (2)	−0.006 (2)
C2	0.0444 (17)	0.0383 (17)	0.0393 (16)	−0.0001 (13)	0.0149 (13)	−0.0041 (12)
C3	0.0439 (16)	0.0348 (15)	0.0472 (17)	0.0000 (13)	0.0188 (13)	0.0000 (12)
C4	0.0335 (15)	0.0300 (14)	0.0444 (16)	−0.0032 (12)	0.0114 (12)	−0.0044 (12)
C5	0.0441 (19)	0.075 (3)	0.053 (2)	0.0015 (18)	0.0036 (16)	0.0057 (18)
C6	0.0394 (16)	0.0485 (19)	0.0436 (17)	−0.0009 (14)	0.0163 (14)	−0.0008 (14)
C7	0.0530 (19)	0.0474 (19)	0.0449 (17)	−0.0104 (15)	0.0013 (15)	0.0036 (15)
C8	0.0409 (16)	0.0355 (16)	0.0468 (17)	−0.0046 (13)	0.0122 (14)	−0.0004 (13)
C9	0.050 (2)	0.060 (2)	0.075 (2)	0.0021 (17)	0.0360 (18)	0.0016 (18)
C10	0.0316 (15)	0.0415 (17)	0.0423 (16)	0.0022 (13)	0.0072 (12)	−0.0005 (13)
C11	0.065 (2)	0.0460 (19)	0.0474 (19)	0.0052 (16)	0.0111 (16)	0.0047 (15)
C12	0.0385 (15)	0.0337 (16)	0.0499 (17)	0.0005 (12)	0.0191 (13)	0.0033 (13)
C13	0.0322 (14)	0.0293 (14)	0.0408 (15)	−0.0007 (11)	0.0108 (12)	−0.0010 (11)

### Geometric parameters (Å, °)

**Table:**

O1—C2	1.339 (4)	C5—H5B	0.9600
O1—C1	1.430 (4)	C5—H5C	0.9600
O2—C2	1.202 (4)	C6—C7	1.491 (4)
O3—C6	1.197 (4)	C7—C8	1.519 (4)
O4—C6	1.331 (4)	C7—H7A	0.9700
O4—C5	1.436 (4)	C7—H7B	0.9700
O5—C10	1.209 (4)	C8—C13	1.538 (4)
O6—C12	1.206 (4)	C8—H8A	0.9700
C1—H1A	0.9600	C8—H8B	0.9700
C1—H1B	0.9600	C9—C10	1.494 (4)
C1—H1C	0.9600	C9—H9A	0.9600
C2—C3	1.495 (4)	C9—H9B	0.9600
C3—C4	1.524 (4)	C9—H9C	0.9600
C3—H3A	0.9700	C10—C13	1.540 (4)
C3—H3B	0.9700	C11—C12	1.501 (5)
C4—C13	1.539 (4)	C11—H11A	0.9600
C4—H4A	0.9700	C11—H11B	0.9600
C4—H4B	0.9700	C11—H11C	0.9600
C5—H5A	0.9600	C12—C13	1.546 (4)
			
C2—O1—C1	116.4 (3)	C8—C7—H7A	108.9
C6—O4—C5	117.5 (3)	C6—C7—H7B	108.9
O1—C1—H1A	109.5	C8—C7—H7B	108.9
O1—C1—H1B	109.5	H7A—C7—H7B	107.7
H1A—C1—H1B	109.5	C7—C8—C13	113.8 (2)
O1—C1—H1C	109.5	C7—C8—H8A	108.8
H1A—C1—H1C	109.5	C13—C8—H8A	108.8
H1B—C1—H1C	109.5	C7—C8—H8B	108.8
O2—C2—O1	122.5 (3)	C13—C8—H8B	108.8
O2—C2—C3	125.8 (3)	H8A—C8—H8B	107.7
O1—C2—C3	111.8 (3)	C10—C9—H9A	109.5
C2—C3—C4	111.6 (2)	C10—C9—H9B	109.5
C2—C3—H3A	109.3	H9A—C9—H9B	109.5
C4—C3—H3A	109.3	C10—C9—H9C	109.5
C2—C3—H3B	109.3	H9A—C9—H9C	109.5
C4—C3—H3B	109.3	H9B—C9—H9C	109.5
H3A—C3—H3B	108.0	O5—C10—C9	120.6 (3)
C3—C4—C13	114.8 (2)	O5—C10—C13	121.5 (3)
C3—C4—H4A	108.6	C9—C10—C13	117.9 (3)
C13—C4—H4A	108.6	C12—C11—H11A	109.5
C3—C4—H4B	108.6	C12—C11—H11B	109.5
C13—C4—H4B	108.6	H11A—C11—H11B	109.5
H4A—C4—H4B	107.5	C12—C11—H11C	109.5
O4—C5—H5A	109.5	H11A—C11—H11C	109.5
O4—C5—H5B	109.5	H11B—C11—H11C	109.5
H5A—C5—H5B	109.5	O6—C12—C11	121.3 (3)
O4—C5—H5C	109.5	O6—C12—C13	121.1 (3)
H5A—C5—H5C	109.5	C11—C12—C13	117.5 (3)
H5B—C5—H5C	109.5	C8—C13—C4	113.4 (2)
O3—C6—O4	123.0 (3)	C8—C13—C10	108.4 (2)
O3—C6—C7	125.7 (3)	C4—C13—C10	108.9 (2)
O4—C6—C7	111.2 (3)	C8—C13—C12	109.4 (2)
C6—C7—C8	113.3 (3)	C4—C13—C12	109.2 (2)
C6—C7—H7A	108.9	C10—C13—C12	107.3 (2)
			
C1—O1—C2—O2	−2.7 (5)	C3—C4—C13—C10	170.5 (2)
C1—O1—C2—C3	177.0 (3)	C3—C4—C13—C12	53.6 (3)
O2—C2—C3—C4	−5.9 (4)	O5—C10—C13—C8	−118.2 (3)
O1—C2—C3—C4	174.3 (3)	C9—C10—C13—C8	61.1 (3)
C2—C3—C4—C13	175.5 (2)	O5—C10—C13—C4	5.6 (4)
C5—O4—C6—O3	0.9 (5)	C9—C10—C13—C4	−175.1 (3)
C5—O4—C6—C7	−177.8 (3)	O5—C10—C13—C12	123.7 (3)
O3—C6—C7—C8	4.6 (5)	C9—C10—C13—C12	−57.0 (3)
O4—C6—C7—C8	−176.7 (3)	O6—C12—C13—C8	9.0 (4)
C6—C7—C8—C13	−171.8 (3)	C11—C12—C13—C8	−172.9 (3)
C7—C8—C13—C4	−66.3 (3)	O6—C12—C13—C4	−115.7 (3)
C7—C8—C13—C10	54.8 (3)	C11—C12—C13—C4	62.4 (3)
C7—C8—C13—C12	171.5 (3)	O6—C12—C13—C10	126.4 (3)
C3—C4—C13—C8	−68.8 (3)	C11—C12—C13—C10	−55.5 (3)

### Hydrogen-bond geometry (Å, °)

**Table:**

*D*—H···*A*	*D*—H	H···*A*	*D*···*A*	*D*—H···*A*
C4—H4B···O5^i^	0.97	2.58	3.418 (4)	145
C3—H3B···O2^ii^	0.97	2.55	3.500 (4)	165

Symmetry codes: (i) −*x*+2, −*y*, −*z*+2; (ii) −*x*+2, *y*−1/2, −*z*+3/2.
